# Enhancing the Cycling Stability of Amorphous-Based LiNiO_2_–Li_2_MnO_3_–Li_2_SO_4_ Positive Electrodes: Insights from In Situ Transmission Electron Microscopy

**DOI:** 10.1021/acsnano.5c17388

**Published:** 2025-11-24

**Authors:** Yuki Nomura, Daiki Hiraoka, Kazuo Yamamoto, Tsukasa Hirayama, Kota Motohashi, Atsushi Sakuda, Akitoshi Hayashi

**Affiliations:** † Nanostructures Research Laboratory, 92043 Japan Fine Ceramics Center, 2−4−1 Mutsuno, Atsuta-ku,, Nagoya 456-8587, Aichi, Japan; ‡ Department of Applied Chemistry, Graduate School of Engineering, 12936 Osaka Metropolitan University, 1−1 Gakuen-cho, Naka-ku, Sakai, Osaka 599-8531, Japan

**Keywords:** scanning transmission electron microscopy, electron energy-loss spectroscopy, nanoparticles, amorphous matrix, Li-rich positive electrodes, flexible volume change

## Abstract

Li-rich layered oxides offer high capacities for solid-state batteries. However, they suffer from sluggish ion transport, interfacial side reactions, and structural instability. Herein, we address these limitations using an amorphous-based LiNiO_2_–Li_2_MnO_3_–Li_2_SO_4_ positive electrode that couples an S-enriched amorphous matrix with Ni-enriched nanocrystalline domains. A two-step process, in which LiNiO_2_ and Li_2_MnO_3_ react prior to the addition of Li_2_SO_4_, suppresses Ni/Mn segregation and eliminates highly oxidized Li–Ni–O domains formed during charge–discharge reactions. In situ scanning transmission electron microscopy with electron energy-loss spectroscopy directly visualizes the evolution of the Li distribution and electronic states of O and Ni during cycling, confirming that both the nanoparticles and the amorphous matrix are electrochemically active, whereas localized deep charging is absent. The ductile amorphous–crystalline framework accommodates reversible volume changes without cracking and interfacial delamination. Consequently, sulfide-based solid-state cells obtained using the two-step synthesis route delivered 173 mAh/g initially and retained 79% capacity after 300 cycles at an aggressive 4.6 V vs Li cutoff voltage without any positive electrode coating. These findings demonstrate that cation homogeneity, nanoscale particle-size control, and flexibility provided by the amorphous matrix act synergistically to enhance the potential of Li-rich positive electrodes in solid-state batteries. The synthesis strategy and mechanistic insights presented herein provide a clear roadmap for the design of robust, high-capacity amorphous-based positive electrodes with extended lifetimes.

## Graphical abstract

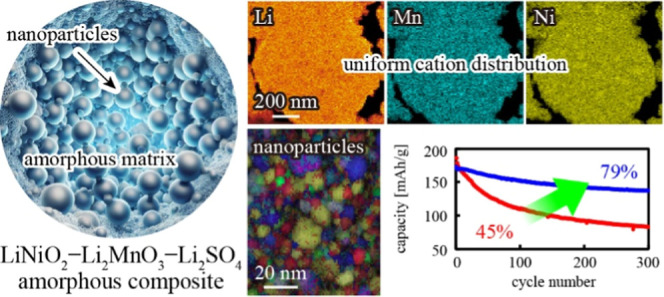

Solid-state Li batteries have attracted significant attention because of their potential for achieving improved energy density and safety compared with the conventional Li batteries based on liquid electrolytes.^1,2^ However, the realization of high-performance solid-state batteries requires positive electrode materials that offer both high capacity and robust long-term cycling stability. Li-rich layered oxides are highly promising candidates owing to their high operating voltage and capacity.^3–7^ In particular, batteries including Li-rich layered positive electrodes and sulfide solid electrolytes have been explored; however, it was found that sluggish transport kinetics and severe interfacial degradation sharply restrict the deliverable capacity, posing a major obstacle to their practical implementation.^8,9^ To address these issues, an amorphous-based LiNiO_2_–Li_2_MnO_3_–Li_2_SO_4_ material has been proposed that incorporates Li_2_SO_4_ to form a composite material consisting of nanoparticles dispersed in an amorphous matrix.^10–12^ This material is designed to offer high capacity by exploiting the Ni- and O-based redox couples provided by the LiNiO_2_–Li_2_MnO_3_ nanoparticles, together with the mechanical flexibility provided by Li_2_SO_4_.^13,14^ In this approach, the amorphous Li_2_SO_4_ matrix functions as a coating layer on the positive electrode materials and mitigates electrochemical degradation at the interface with the sulfide solid electrolyte.^15–17^ Moreover, the high ductility of the proposed amorphous–crystalline composite structure is expected to enable intimate contact with the solid electrolyte, thereby enhancing ionic transport and lowering interfacial resistance over repeated cycles. The Li_2_SO_4_ component acts as an “inorganic binder (softening agent)” within the composites. Owing to its low melting point, it accommodates the volumetric expansion and contraction associated with (de)­lithiation, contributing to the long-term stability of the composites.^18^ Furthermore, the introduction of Li_2_SO_4_ can improve the ionic conductivity, helping to overcome the sluggish transport kinetics in the positive electrode layer.

However, these attributes remain theoretical design goals and are yet to be fully validated by experimental evidence. Despite their potential advantages, the amorphous-based LiNiO_2_–Li_2_MnO_3_–Li_2_SO_4_ positive electrode materials exhibit substantial capacity fading under repeated cycling, highlighting the need for further durability improvements.^12^ The inherently complex structure, composed of an amorphous matrix interspersed with nanoscale crystalline domains, is a key factor underlying these challenges.^19^ Moreover, due to the complex structure, the specific contributions of each structural component to the electrochemical reactions remain unclear. The evolution of local electrochemistry, and in particular the distributions of Li, Ni, Mn, S, and O and corresponding changes in their electronic states, remain insufficiently understood. Addressing these knowledge gaps is critical for elucidating the complete charge–discharge mechanism and developing strategies to mitigate the degradation processes.

In the present work, we report an improved synthesis strategy for the amorphous-based LiNiO_2_–Li_2_MnO_3_–Li_2_SO_4_ positive electrode materials that effectively suppresses local Ni and Mn inhomogeneities, resulting in enhanced cycle performance compared with the earlier variants of these positive electrode materials. To gain deeper insight into the operation of these improved materials during charging and discharging, we employed in situ scanning transmission electron microscopy (STEM) combined with electron energy-loss spectroscopy (EELS),^20–23^ enabling real-space observation of the changes in the Li distribution and the electronic states of O and Ni at the nanometer scale. This technique enabled the visualization of how variations in the synthesis process affect the nanostructures and electrochemical reactions, and allowed determination of whether the reactions during charging and discharging involve nanosized crystallites, amorphous matrix, or both. We also observed flexible expansion-contraction behavior arising from the intrinsically high ductility of the material, which suppresses crack formation and interfacial delamination from solid electrolytes. The results of this study not only offer a clearer understanding of the fundamental charge–discharge mechanisms in the amorphous-based LiNiO_2_–Li_2_MnO_3_–Li_2_SO_4_ positive electrode materials but also provide key guidelines for engineering robust and high-capacity positive electrode materials featuring the amorphous Li_2_SO_4_ matrix.

## Results and Discussion

We synthesized amorphous-based LiNiO_2_–Li_2_MnO_3_–Li_2_SO_4_ positive electrode materials using two different approaches, namely a single-step and two-step process. The single-step process followed a previously reported method.^12^ Specifically, LiNiO_2_, Li_2_MnO_3_, and Li_2_SO_4_ powders were directly combined in a 60:20:20 molar ratio and a mechanochemical treatment was conducted. The resulting powder was then heat-treated at 300 or 400 °C for 1 h, yielding 60LiNiO_2_·20Li_2_MnO_3_·20Li_2_SO_4_ (referred to as LNMS-622 in this paper). By contrast, the two-step process represents the improved synthesis strategy, where LiNiO_2_ and Li_2_MnO_3_ were first combined in a 60:20 molar ratio and underwent the mechanochemical treatment followed by sintering at 900 °C for 10 h to obtain Li_1.11_Ni_0.67_Mn_0.22_O_2_. This material was then blended with Li_2_SO_4_ in an 80:20 molar ratio and subjected to a mechanochemical treatment. The milled powder was subsequently heat-treated at 300 or 400 °C for 1 h, producing 80Li_1.11_Ni_0.67_Mn_0.22_O_2_·20Li_2_SO_4_ (hereafter LNMS-82). Despite the differences in the synthetic procedures, both methods yielded materials with the same starting composition as the precursor materials.

Figure 1a compares the charge–discharge curves of the solid-state battery cells containing LNMS-622 (prepared via the single-step process) or LNMS-82 (prepared via the two-step process) for 5 cycles within a voltage range of 1.4–4.0 V vs Li–In. (2.0–4.6 V vs Li). These cells were constructed with an argyrodite-type Li_6–*x*_PS_5–*x*_Cl_1+*x*_ solid electrolyte^24,25^ and a Li–In alloy negative electrode. LNMS-622 and LNMS-82 were tested either without annealing or after annealing at 300 or 400 °C. For LNMS-622, the highest initial charge and discharge capacities of 192 and 186 mAh/g, respectively, were achieved by the sample annealed at 300 °C. However, these capacities decreased to 173 and 170 mAh/g, respectively, after 5 cycles. A similar trend was observed for the sample without annealing and that annealed at 400 °C. By contrast, the LNMS-82 sample showed a slightly lower initial capacity than the LNMS-622 sample annealed at 300 °C, but underwent notably lower capacity loss with cycling. Among the LNMS-82 variants, the sample annealed at 400 °C showed the highest initial charge and discharge capacities of 186 and 173 mAh/g, respectively, and retained 172 and 170 mAh/g after 5 cycles. The samples without annealing and those annealed at 300 °C exhibited the same trend. Hereafter, we compared the 300 °C-annealed LNMS-622 and the 400 °C-annealed LNMS-82, each of which demonstrated the highest capacity in its respective series. Figure 1b illustrates the cycling performance of the 300 °C-annealed LNMS-622 and the 400 °C-annealed LNMS-82 over 300 cycles. After 300 cycles, LNMS-622 experienced a drop in the discharge capacity from 186 to 83 mAh/g (45% retention). By contrast, LNMS-82 exhibited an impressive 79% retention, decreasing from 173 to only 137 mAh/g despite the same demanding cutoff voltage, highlighting its substantially improved cycle performance. Supporting Figure S1 shows the rate performance characteristics of LNMS-622 and LNMS-82. Starting from the sixth cycle, LNMS-82 exhibited a higher discharge capacity than LNMS-622 at all tested rates. The values for the capacity retention at 2.5 mA/cm^2^ relative to that at 0.13 mA/cm^2^ were 23% and 43% for LNMS-622 and LNMS-82, respectively. Thus, both the cycling and rate capabilities were enhanced through the optimization of the synthesis process. The X-ray diffraction (XRD) patterns of LNMS-622 and LNMS-82, both unannealed and annealed at 300 and 400 °C, are presented in Supporting Figure S2. Except for the LNMS-622 sample annealed at 400 °C, no substantial differences were evident between the XRD patterns of the various samples. These materials were neither highly crystalline nor amorphous; rather, the mechanochemical treatment disrupted the long-range but not the short-range order, resulting in metastable nanocrystalline domains. The peaks observed at 38°, 44°, and 65° were assigned to the cubic rock-salt structure (Fm3̅m), while the new peak at 22° in the 400 °C-annealed LNMS-622 sample was attributed to Li_2_SO_4_, suggesting that Li_2_SO_4_ underwent crystallization under these annealing conditions. It is difficult to explain the differences in the electrochemical properties based solely on the crystal structures revealed by the XRD measurements.

**Figure 1:**
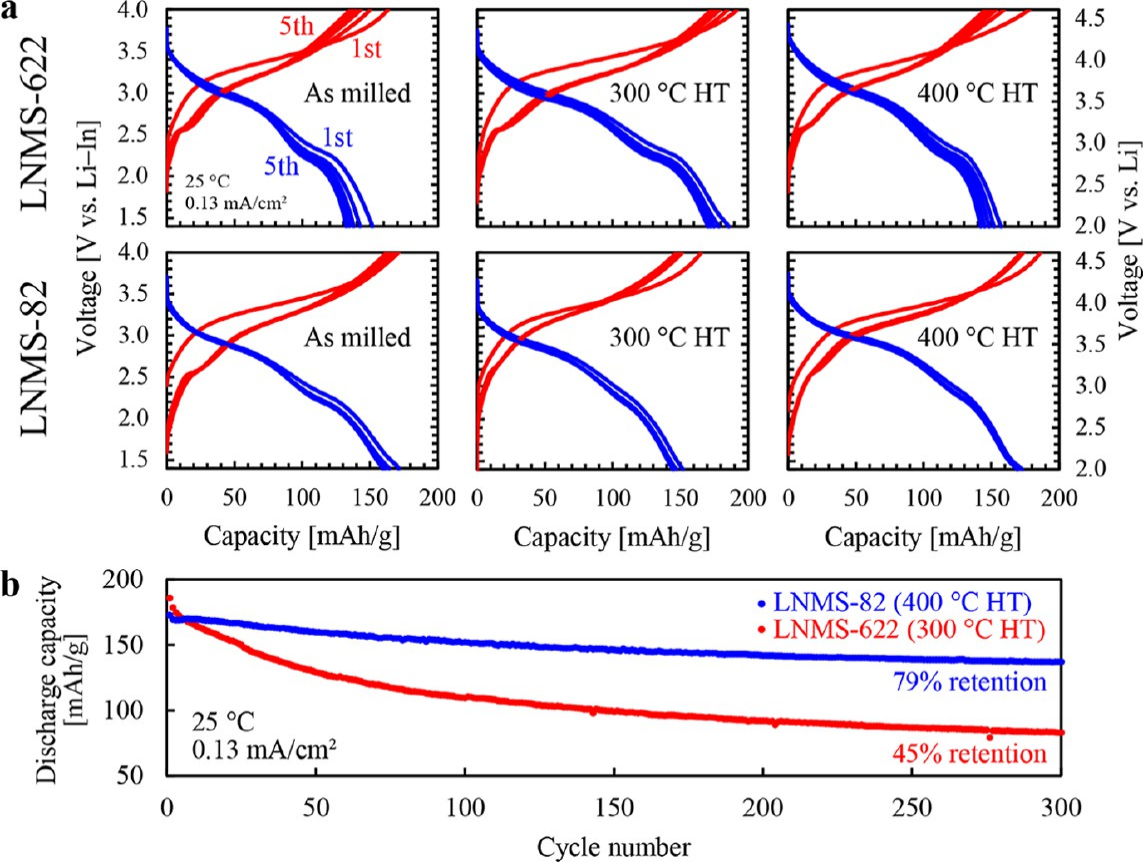
Electrochemical characterization of solid-state batteries using 60LiNiO_2_·20Li_2_MnO_3_·20Li_2_SO_4_ (LNMS-622, prepared via a single-step process) and 80Li_1.11_Ni_0.67_Mn_0.22_O_2_·20Li_2_SO_4_ (LNMS-82, prepared via a two-step process). (a) Charge–discharge curves for the samples obtained without annealing and with annealing at 300 or 400 °C for 5 cycles within a voltage range of 1.4–4.0 V vs Li–In (2.0–4.6 V vs Li). (b) Comparison of the long-term cycling performance for 300 cycles.

We performed ex situ STEM-EELS analysis to investigate the nanostructure of the LNMS-622 sample annealed at 300 °C. Figure 2 shows the positive electrode layer in a solid-state battery cell employing LNMS-622. In the annular dark-field (ADF) STEM image (Figure 2a), the LNMS-622 particles were observed next to the solid electrolyte layer, indicating an intimate interface between the two materials. Figure 2b–e show the distributions of Li, O, Mn, and Ni, revealing large Ni-rich and Mn-poor particles (40–100 nm) coexisting in a more uniformly dispersed matrix, suggesting that LiNiO_2_ and Li_2_MnO_3_ were not fully integrated into the composite. These particles also exhibited relatively low Li concentrations. We quantified the Ni, Mn, and S atomic ratios by energy-dispersive X-ray spectroscopy (EDS). The STEM-EDS analysis (Supporting Figure S3) showed a clear size-dependent compositional contrast. The large nanoparticles exhibited an average atomic ratio of Ni/Mn/S ≈ 92:4:4, whereas the surrounding matrix showed a ratio of Ni/Mn/S ≈ 54:21:25. These results indicate that the large nanoparticles are Li–Ni–O particles, while the surrounding matrix is a well-mixed Li–Ni–Mn–S–O composite. High-resolution TEM image and fast Fourier transform (FFT) patterns (Figure 2f) represent ∼10 nm crystalline domains dispersed within an amorphous matrix. High-magnification STEM-EDS (Supporting Figure S4) showed that the 5–10 nm nanoparticles, which appeared bright in ADF-STEM images, were Ni-enriched, whereas the dark-contrast regions were S-enriched. These results demonstrate that the large Li–Ni–O particles (40–100 nm) and small Ni-enriched nanoparticles (∼10 nm) are dispersed within an S-enriched matrix. To verify the crystal structure across different particle sizes and within the matrix, we acquired precession electron diffraction patterns. Patterns from both nanoparticle size classes were indexed to the Fm3̅m structure, whereas the matrix exhibited no distinct diffraction spots (Supporting Figure S5), confirming that the nanoparticles were crystalline and the matrix was amorphous. The crystal orientation maps (Figure 2g,h) obtained by scanning precession electron diffraction further confirmed that the crystalline domains consisted of distinct nanoparticles, each with its own crystallographic orientation.^26–28^ The nanoparticles in the observed regions ranged from 5 to 70 nm in size. Finally, Figure 2i shows a schematic of the overall nanostructure, consisting of large Li–Ni–O particles (40–100 nm) crystallizing in the Fm3̅m structure, dispersed within an S-enriched amorphous matrix (gray), together with numerous smaller Ni-enriched nanoparticles (5–10 nm, yellow) that also adopt the Fm3̅m structure. This hierarchical structure of the amorphous matrix and nanocrystallites plays a crucial role in the electrochemical properties of LNMS-622.

**Figure 2:**
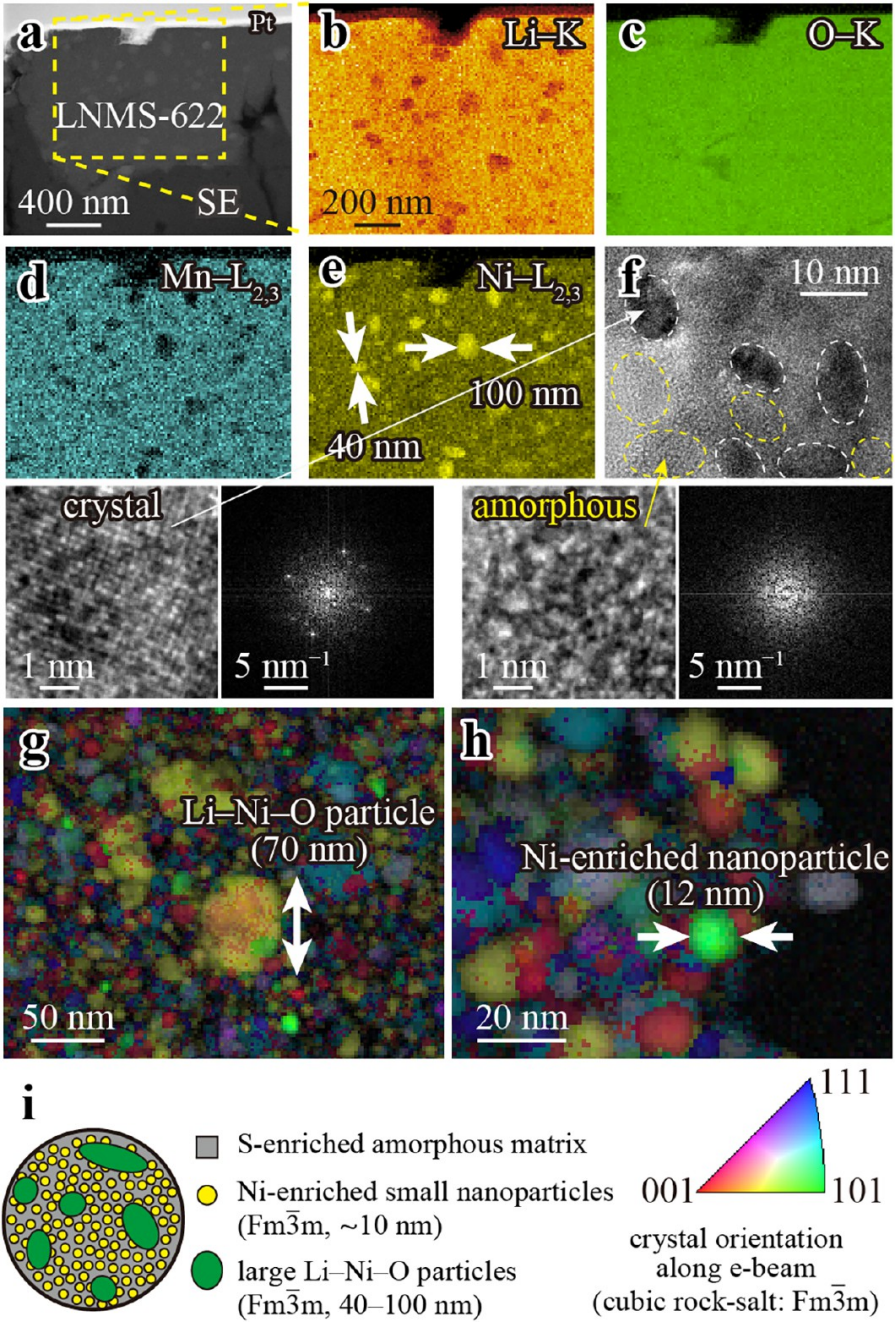
Nanostructure of LNMS-622 annealed at 300 °C. (a) ADF-STEM image of an LNMS-622 particle adjacent to the solid electrolyte layer. (b–e) Elemental maps of Li, O, Mn, and Ni, revealing the presence of large Ni-rich and Mn-poor particles (40–100 nm). (f) High-resolution TEM image. Dashed white circles indicate ∼10 nm crystalline domains, whereas dashed yellow circles highlight the amorphous matrix. The lower panels present magnified views of these crystalline and amorphous regions along with their corresponding FFT patterns. (g,h) Crystal orientation maps of the crystalline domains, indicating the presence of distinct nanoparticles. (i) Schematic of the overall nanostructure, showing the S-enriched amorphous matrix (gray), small Ni-enriched nanoparticles (Fm3̅m, ∼10 nm, yellow), and large Li–Ni–O particles (Fm3̅m, 40–100 nm, green).

To elucidate how the hierarchical structure of the amorphous matrix and nanocrystallites affects the electrochemical properties, we performed in situ STEM-EELS analysis on 300 °C-annealed LNMS-622 under 4.0 V vs Li–In charging and 1.4 V vs Li–In discharging at room temperature. Details of the in situ biasing setup, including the battery configuration and electrical-contact geometry, are provided in Supporting Figure S6 and in the Experimental section. Figure 3a shows the EEL spectra before and after charging. The Li K-edge intensity decreased during charging, indicating the extraction of Li ions from LNMS-622. Concurrently, the prepeak in the O–K edge at ∼529 eV became more pronounced, suggesting that electrons were removed from the orbitals formed by hybridization between Ni-3d and O-2p,^29,30^ because removal of these electrons increased the probability of the electron transition from O-1s to O-2p in the EELS measurements. Therefore, the intensity of the O–K prepeak reflected the population of ligand holes in the hybridized orbitals.^31^ Moreover, the Ni−L_3_ peak shifted toward higher energies, reflecting the oxidized electronic state of Ni after charging. Although a slight shift was observed at the Mn−L_3_ edge, its magnitude was significantly smaller, indicating that the electrochemical contribution of Mn was considerably lower than that of Ni. EELS can, in principle, provide quantitative atomic concentrations or ratios from core-loss spectra. However, increasing the specimen thickness raises the probability of plural scattering and thereby reduces quantitative accuracy. Errors in elemental ratios become significant when *t*/λ (where *t* is the local thickness and λ is the inelastic mean free path) exceeds ∼0.5.^32^ In in situ biasing experiments, both electronic and ionic conduction pathways must remain intact. Consequently, the field of view must cover a relatively wide, micrometer-scale region. Thinning such an extended area uniformly to below 0.5λ is challenging with current FIB techniques. As a result, the region examined in this study had an average *t*/λ ∼0.6–1.0, which was beyond the range for reliable quantification. We therefore adopted a qualitative approach, mapping the integrated intensity of the Li–K edge rather than attempting absolute concentration analysis.

**Figure 3:**
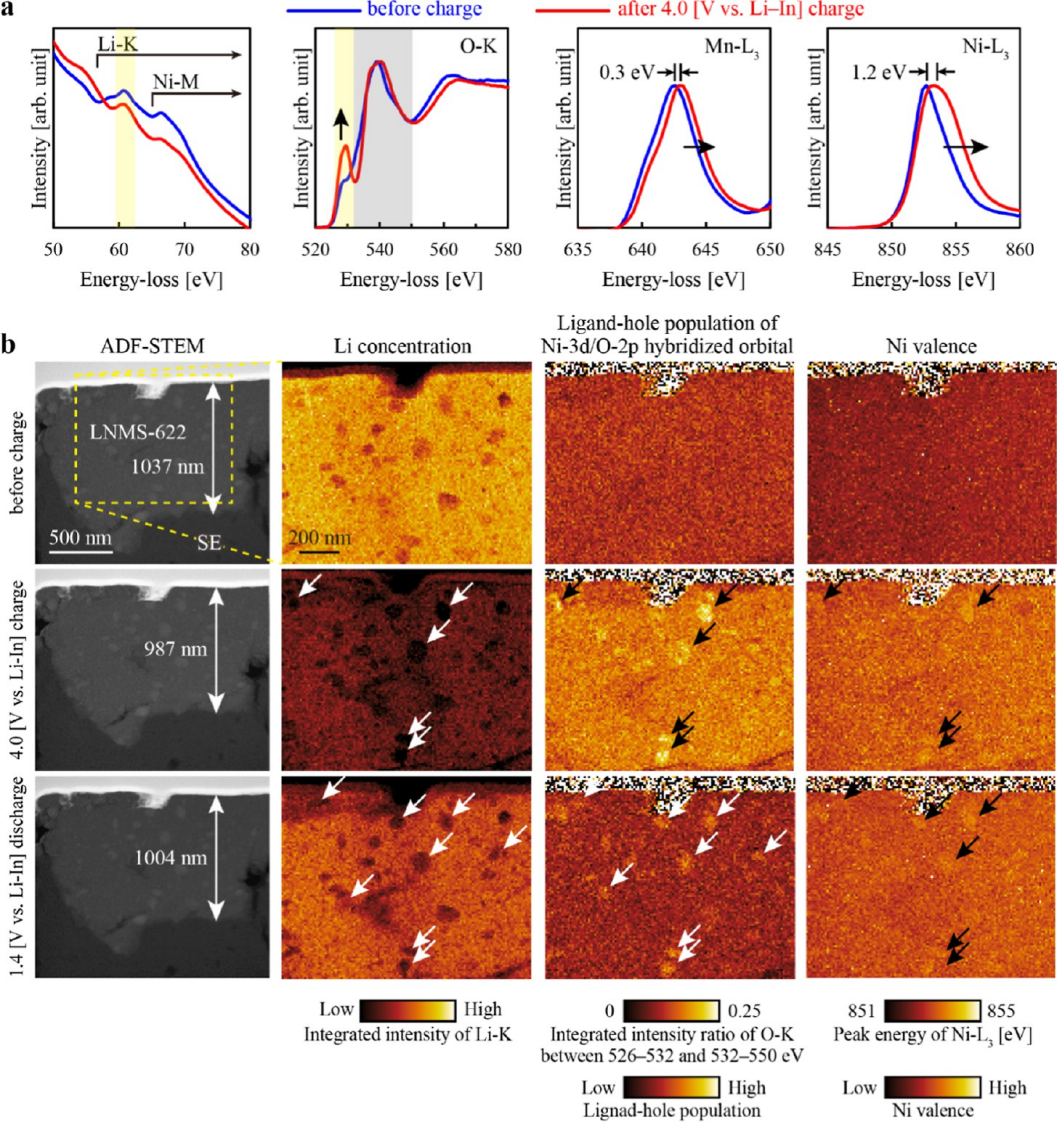
In situ STEM-EELS analysis of LNMS-622 annealed at 300 °C. (a) Li–K, O–K, Mn−L_3_, and Ni−L_3_ EEL spectra before and after charging. The O–K prepeak intensity at ∼529 eV reflects the ligand-hole population of the Ni-3d/O-2p hybridized orbitals. The Ni−L_3_ peak energy reflects the Ni valence. (b) Changes in the ADF-STEM image, Li distribution, O–K integrated intensity ratio [526–532 (yellow)/532–550 eV (gray)], and Ni−L_3_ peak energy distribution. Black and white arrows indicate large Li–Ni–O nanoparticles. For Li–K edge mapping, the signal was integrated over the 59.5–62.5 eV window (yellow), which lies well below the Ni−M edge (>68 eV),^33^ thereby minimizing contributions from Ni. Self-normalization of the O–K edge was applied to compensate for specimen-thickness variations.

Figure 3b shows the changes in the ADF-STEM image, Li distribution, integrated intensity distribution of the O–K prepeak at ∼529 eV, and peak energy distribution of Ni−L_3_ during charging and discharging. The O–K prepeak intensity (526–532 eV) was normalized to the integrated O–K edge intensity (532–550 eV) to compensate for variations in specimen thickness. The ADF-STEM images showed that the LNMS-622 particles first contracted and then slightly expanded when Li ions were deintercalated and intercalated, respectively. Their height decreased from 1037 to 987 nm and then increased to 1004 nm. However, large cracks and interface delamination were not observed, suggesting that the high ductility of the amorphous–crystalline composite structure enabled flexible contraction and expansion processes. For reference, Supporting Figure S7 shows ADF-STEM images of a LiNi_0.5_Co_0.2_Mn_0.3_O_2_ particle before and after charging at 3.9 V vs Li–In. These images display significant cracking along the grain boundaries of the polycrystalline particle. This comparison underscores the enhanced ductility of the LNMS composite which allows it to more flexibly accommodate volume changes, thereby preventing mechanical degradation.

Next, we examined the changes in the Li distribution and electronic states of O and Ni during charging and discharging. Before charging, the Li distribution was nonuniform, whereas the electronic states of O and Ni were uniform. As shown in Figure 2b–e, the regions with low Li concentrations were Li–Ni–O particles. After charging, Li extraction proceeded in a manner that preserved the initial distribution pattern, causing a pronounced decrease in the Li concentration inside the large Li–Ni–O particles (indicated by white arrows). Across the particles, the ligand-hole population in the Ni-3d/O-2p hybridized orbitals increased, and Ni shifted to a higher oxidation state upon charging (the entire region appeared brighter). However, these changes were most pronounced within large Li–Ni–O particles (indicated by black arrows). After discharging, the Li distribution pattern was similar to those observed initially and after charging. Nevertheless, although the matrix region exhibited a substantial recovery in Li content, recovering 56% of its Li–K intensity, the large Li–Ni–O nanoparticles showed less Li insertion with recovery of only 46%, indicating that fewer Li ions were reinserted into the nanoparticles than that into the matrix. As evidence, the ligand-hole population and Ni valence in these particles remained in a higher oxidation states even after discharging (indicated by white arrows in the ligand-hole population map and black arrows in the Ni valence map), demonstrating incomplete reversibility. These deep charged states in large Li–Ni–O particles can lead to structural and electrochemical instabilities such as large volume changes and potential O loss, which can accelerate degradation over repeated cycles. The tendency toward higher oxidation states observed in the larger Li–Ni–O particles indicates that the enhanced dispersion of Ni and Mn is important for improving cycling stability. It should be noted that our previous operando STEM-EELS measurements using the same setup revealed that (de)­lithiation commenced immediately after the reaction started—even in FIB-thinned regions (100–200 nm thick)—indicating that thinning did not significantly impede the reaction kinetics.^23^ In the present study, we also applied a 1 h constant-voltage hold after each galvanostatic (dis)­charge to homogenize the Li distribution, thereby ensuring uniform Li distribution in both thin and thick regions of the battery. This procedure confirmed that the observed changes represented intrinsic LNMS behavior rather than artifacts associated with lamella geometry.

Next, we present the results of the nanostructural analysis of LNMS-82 annealed at 400 °C to investigate the influence of the changes in the synthesis process on its nanostructure. Figure 4a–e show an ADF-STEM image and the elemental distributions of Li, O, Mn, and Ni for the LNMS-82 particle. In contrast to that in LNMS-622, no Ni-rich or Mn-poor regions were apparent, indicating that Ni and Mn were more uniformly distributed because of the prereaction of LiNiO_2_ and Li_2_MnO_3_. However, the composition was not entirely uniform. In particular, large nanoparticles (∼50 nm) were observed as indicated by the dashed circle in the ADF-STEM image and the Li, Mn, and Ni elemental maps. Figure 4f shows a high-resolution TEM image and FFT patterns of the crystalline and amorphous regions, revealing ∼10 nm crystalline domains dispersed within an amorphous matrix. Similar to LNMS-622, numerous nanoparticles were dispersed throughout the sample. The crystal orientation maps (Figure 4g,h) showed that these crystalline domains were composed of distinct nanoparticles, each with its own crystallographic orientation. The nanoparticle sizes ranged from 5 to 45 nm in the observed regions. It is important to note that the 45 nm particles were not Li–Ni–O domains; rather, they contained both Ni and Mn, as shown in Figure 4d,e. Quantitative STEM-EDS mapping (Supporting Figure S8) showed that the large nanoparticles in LNMS-82 had a composition of Ni/Mn/S ≈ 64:22:14. Compared with LNMS-622 (Ni/Mn/S ≈ 92:4:4), LNMS-82 is less Ni-enriched. Furthermore, high-magnification STEM-EDS (Supporting Figure S9) showed that, similar to LNMS-622, the 5–10 nm nanoparticles were Ni-enriched, while the matrix was S-enriched. These findings indicate that Ni-enriched nanoparticles, exhibiting a size distribution, are embedded within an S-enriched matrix. Precession electron diffraction confirmed that nanoparticles of all sizes adopted the Fm3̅m lattice, while the matrix exhibited no distinct diffraction spots (Supporting Figure S10). Figure 4i shows a schematic summarizing the overall nanostructure of LNMS-82 annealed at 400 °C: numerous Ni-enriched nanoparticles (yellow, 5–50 nm) crystallizing in the Fm3̅m structure, dispersed within an S-enriched amorphous matrix (gray).

**Figure 4:**
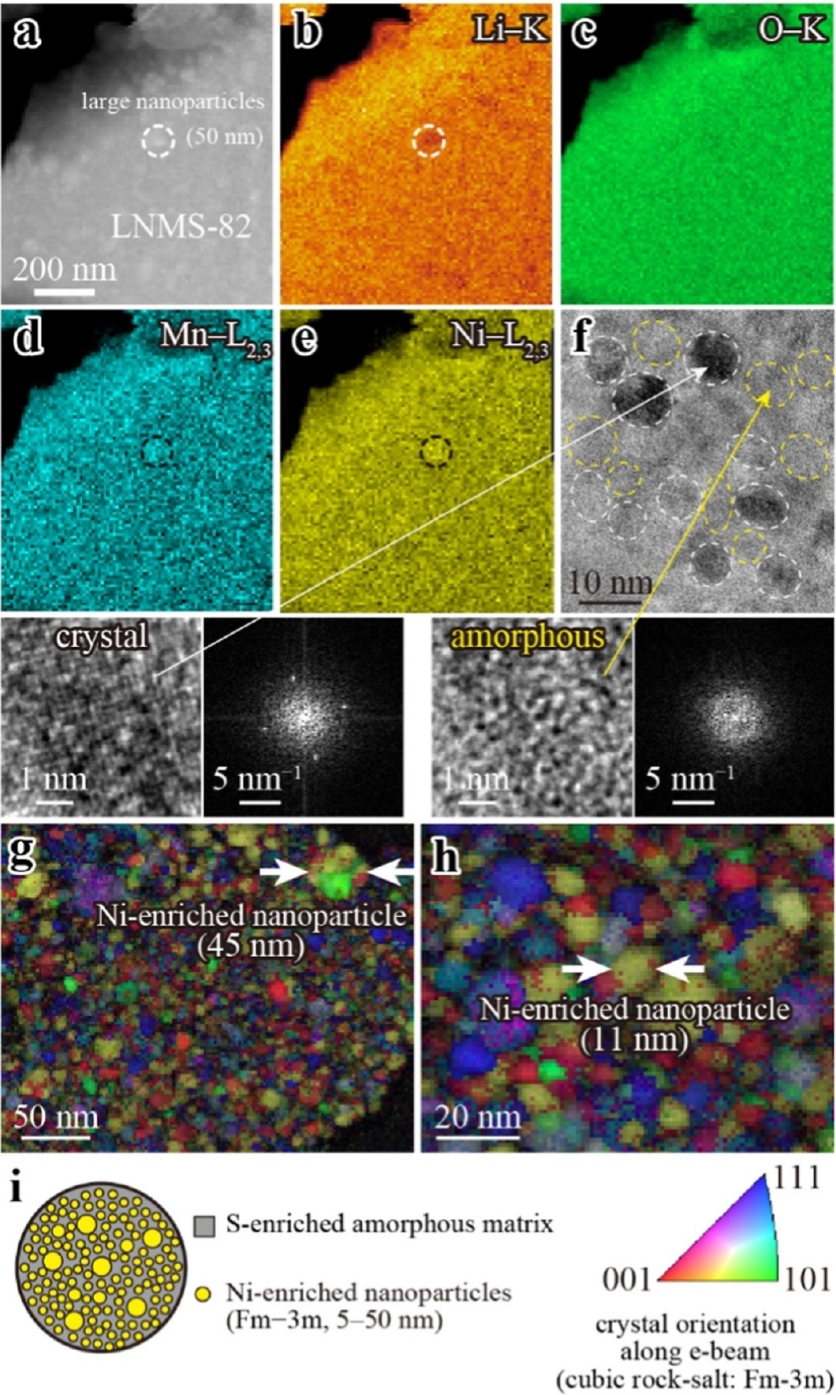
Nanostructure of LNMS-82 annealed at 400 °C. (a) ADF-STEM image. (b–e) Elemental maps of Li, O, Mn, and Ni, revealing the absence of large Li–Ni–O particles. (f) High-resolution TEM image, highlighting nanosized crystallites. White dashed circles indicate ∼10 nm crystalline domains, while yellow dashed circles highlight the amorphous matrix. The lower panels present magnified views of these crystalline and amorphous regions along with their corresponding FFT patterns. (g,h) Crystal orientation maps of the crystalline domains, indicating the presence of distinct nanoparticles. (i) Schematic of the overall nanostructure, showing the S-enriched amorphous matrix (gray) and Ni-enriched nanoparticles (Fm3̅m, 5–50 nm, yellow).

To investigate the influence of these nanostructures on the electrochemical behavior, we carried out in situ STEM-EELS analysis on LNMS-82 annealed at 400 °C. Figure 5a presents the EEL spectra of LNMS-82 before and after charging. The changes in the Li–K, O–K, Mn−L_3_, and Ni−L_3_ edges resembled those observed for LNMS-622. Li extraction decreased the Li–K intensity, increased the O–K pre-edge intensity, and shifted the Ni−L_3_ edge to higher energy, indicating Li depletion, an increased ligand-hole population, and a higher Ni valence state. Figure 5b presents the results of the in situ observations of LNMS-82 under charging to 4.0 V vs Li–In and discharging to 1.4 V vs Li–In at room temperature, highlighting changes in the ADF-STEM image, Li distribution, ligand-hole population in the Ni-3d/O-2p hybridized orbitals, and Ni valence state. The spatial step size was 10 nm, consistent with that used in Figure 3b. The ADF-STEM images obtained after charging and discharging showed that the LNMS-82 particles shrank during Li extraction and then underwent an expansion upon Li insertion. The particle height decreased from 1436 to 1409 nm, followed by an increase to 1433 nm. Similar to LNMS-622, neither interface delamination nor cracking were observed, indicating flexible expansion-contraction behavior. Before charging, the regions with low Li concentrations were not Li–Ni–O particles but rather contained both Ni and Mn. The ligand-hole population in the Ni-3d/O-2p hybridized orbitals and the Ni valence were uniform. After charging, the Li concentration decreased throughout the particles, while both the ligand-hole population and the Ni valence increased, making the entire region appear brighter. In regions containing large nanoparticles (indicated by the black arrow), the change in the ligand-hole population was more pronounced. However, because both the number and size of the large nanoparticles were reduced, regions with high oxidation states were less pronounced than those in LNMS-622. Upon discharging, the Li concentration recovered, and the ligand-hole population and Ni valence decreased. Notably, the high-valence regions observed in LNMS-622 (Figure 3b) were considerably less pronounced in LNMS-82, even after discharge. By eliminating these Li–Ni–O high-valence domains, LNMS-82 maintained its structural stability during prolonged cycling. However, slightly more oxidized particles were observed at the positions of the large Ni-enriched particles indicated by the white arrow, suggesting that reducing the particle size in LNMS-82 could improve cycling performance. In our setup, electrochemical impedance spectra could be acquired inside a transmission electron microscope while simultaneously performing charge–discharge cycling and TEM observations. An example for the solid-state battery using LNMS-82 is shown in Supporting Figure S11. Moving forward, we aim to correlate the resistance values measured by electrochemical impedance spectroscopy with the corresponding structural features and further advance such integrated studies.

**Figure 5:**
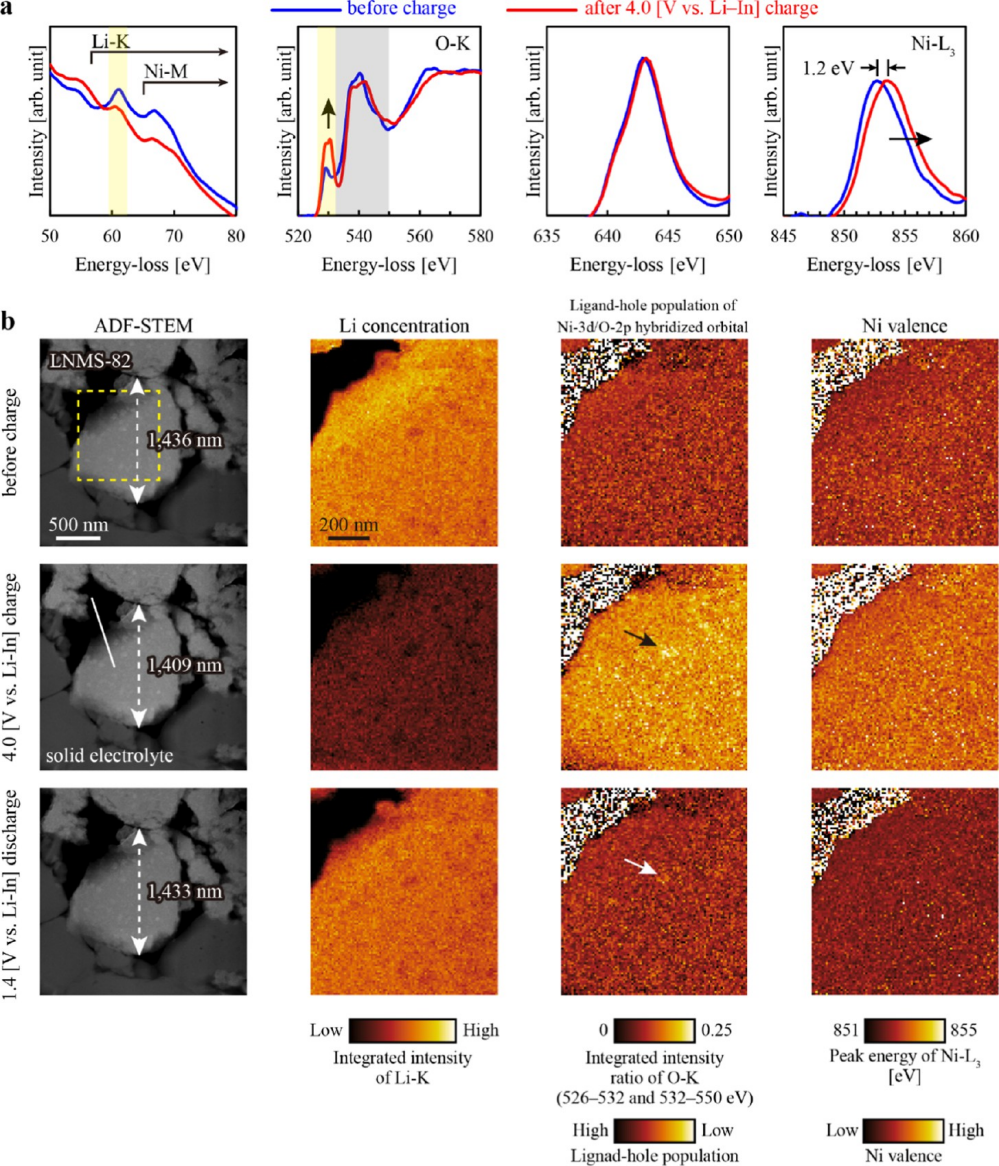
In situ STEM-EELS analysis of LNMS-82 annealed at 400 °C. (a) Li–K, O–K, Mn−L_3_, and Ni−L_3_ EEL spectra before and after charging. (b) Changes in the ADF-STEM image, Li distribution, O–K integrated intensity ratio [526–532 (yellow)/532–550 eV (gray)], and Ni−L_3_ peak energy distribution. Black and white arrows indicate large Ni-enriched nanoparticles (∼50 nm). For Li–K edge mapping, the signal was integrated over the 59.5–62.5 eV window (yellow), which lies well below the Ni-M edge (>68 eV),^33^ thereby minimizing contributions from Ni. Self-normalization of the O–K edge was applied to compensate for specimen-thickness variations.

Finally, to elucidate the contributions of the 5–10 nm Ni-enriched nanoparticles and S-enriched matrix to the electrochemical reaction in LNMS-82, we performed detailed ADF-STEM observations and Li mapping at high magnification (Figure 6) acquired from the same field of view. Notably, this region did not contain any large nanoparticles (∼50 nm), allowing for a more detailed observation of the smaller nanodomains and surrounding matrix. The ellipses superimposed on the images denote areas of different ADF-STEM intensities: black solid ellipses highlight brighter regions containing Ni-enriched nanoparticles, whereas white dashed ellipses indicate darker regions associated with the S-enriched amorphous matrix (Figure S9). Before charging, the matrix exhibited a higher Li concentration than the nanoparticle regions, reflecting the higher Li content in Li_2_SO_4_ relative to LiNiO_2_. While it may be assumed that only nanoparticles participated in the reaction, changes in the Li concentration were detected in both the nanoparticles and the matrix during charging and discharging. Notably, the nanoparticles appeared to overlap in the direction of the electron beam because the thickness of the TEM sample (∼100 nm) exceeded the scale of the nanostructure. Nevertheless, if only the nanoparticles contributed to the reaction, a larger Li concentration difference between the nanoparticles and matrix would be expected after charging. However, no such pronounced difference was observed in the Li distribution after charging; instead, an overall decrease in Li concentration was evident. This showed that both the nanoparticles and the matrix participated in the electrochemical reaction, jointly contributing to the high capacity of the amorphous-based LiNiO_2_–Li_2_MnO_3_–Li_2_SO_4_ positive electrode materials. It should be noted that the white dashed ellipses were not composed solely of Li_2_SO_4_. The STEM-EDS analysis (Supporting Figure S9) confirmed that, in addition to S, Ni and Mn were present in the matrix region. The active Ni component accounted for the observed Li loss, whereas the Li_2_SO_4_ fraction was expected to remain unchanged.^34^

**Figure 6:**
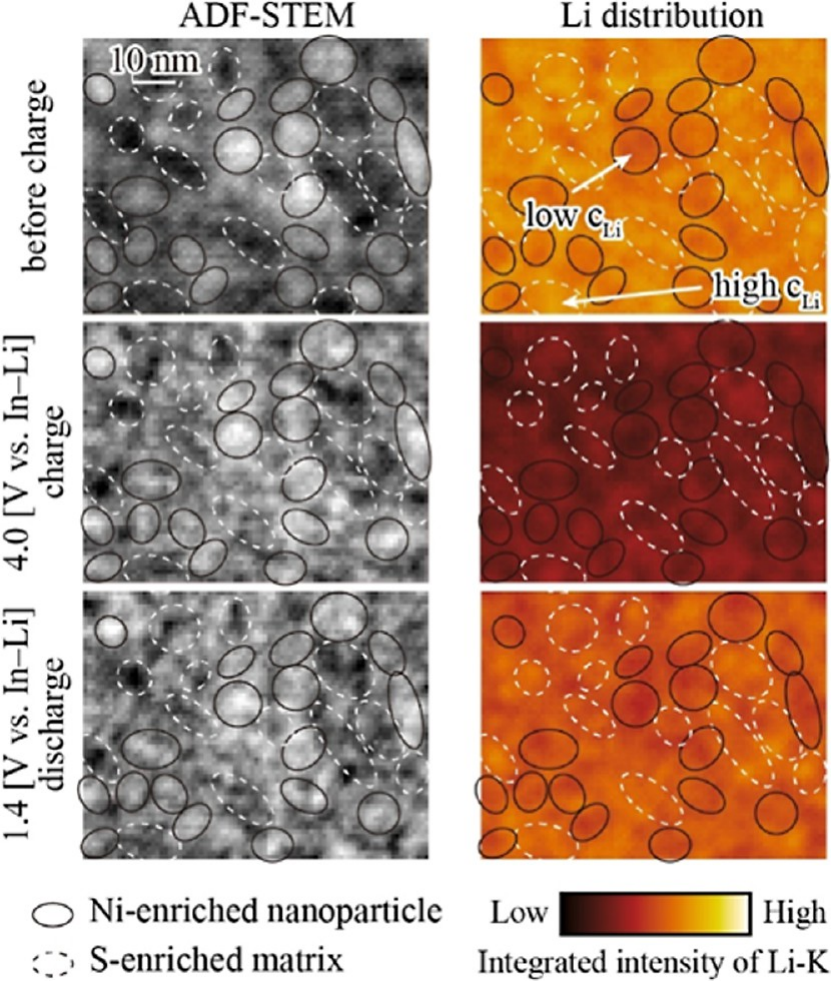
High-magnification in situ STEM-EELS analysis of LNMS-82 annealed at 400 °C. Ellipses denote regions of differing intensities in the ADF-STEM images. Black solid ellipses highlight high-intensity areas corresponding to Ni-enriched nanoparticles, whereas white dashed ellipses indicate low-intensity areas representing the S-enriched matrix.

## Conclusion

We investigated two synthesis routes for amorphous-based LiNiO_2_–Li_2_MnO_3_–Li_2_SO_4_ positive electrode materials, denoted as LNMS-622 (prepared via a single-step process) and LNMS-82 (prepared via a two-step process), and examined their structural and electrochemical properties. Our findings revealed that achieving improved Ni and Mn homogeneities is crucial for enhancing cycle stability: compared with LNMS-622, LNMS-82 showed a more uniform distribution of Ni and Mn and exhibited an improved capacity retention of 79% after 300 cycles at an aggressive cutoff voltage of 4.0 V vs Li–In (4.6 V vs Li) in sulfide-based solid-state batteries without any positive electrode coating. In situ STEM-EELS revealed that LNMS-82 lacked the highly oxidized Li–Ni–O domains observed in LNMS-622, thus mitigating the local deep-charging effects and improving its structural and electrochemical stability.^35^ Moreover, the amorphous–crystalline composite structure contributed to high ductility, allowing flexible expansion and contraction without severe mechanical degradation. These combined electrochemical, compositional, and structural advantages led to superior long-term stability. Furthermore, in situ STEM-EELS showed that both the nanoparticles and the matrix participated in charge–discharge reactions, leading to the high capacity of the materials. We further inferred that reducing the fraction of larger (∼50 nm) nanoparticles in LNMS-82 and achieving a more uniform dispersion of smaller (∼10 nm) nanoparticles can further enhance the stability. Overall, our study highlights the importance of controlling the cation distribution, reducing the size of the nanoparticles, and retaining amorphous flexibility to achieve reliable performance in amorphous-based LiNiO_2_–Li_2_MnO_3_–Li_2_SO_4_ positive electrodes. Future work will focus on further optimization of the synthesis process and nanostructures to improve the energy density and extend the cycle life.

## Experimental Section

### Material Synthesis

LiNiO_2_ (Toshima Corp.), Li_2_MnO_3_ (Toshima Corp.), and Li_2_SO_4_ (Sigma-Aldrich Co., LLC, ≥99.99%) were used as the initial precursors. To eliminate residual moisture, each powder was predried under vacuum at 120 °C for 12 h prior to use. We prepared LiNiO_2_–Li_2_MnO_3_–Li_2_SO_4_ active materials by two distinct methods, namely a single-step process in which LiNiO_2_, Li_2_MnO_3_, and Li_2_SO_4_ were mixed directly^12^ and a two-step process in which LiNiO_2_ and Li_2_MnO_3_ were first reacted prior to the introduction of Li_2_SO_4_. A single-step sample was prepared in an Ar-filled glovebox by weighing LiNiO_2_, Li_2_MnO_3_, and Li_2_SO_4_ in a 60:20:20 molar ratio, mixing them in an agate mortar, and subjecting the mixture to a mechanochemical treatment. Specifically, the precursor mixture (0.5 g) was placed in a 45 mL zirconia pot and milled at 370 rpm for 50 h. The milled powder was then heat-treated at 300 and 400 °C for 1 h. The resulting product is denoted as 60LiNiO_2_·20Li_2_MnO_3_·20Li_2_SO_4_ or LNMS-622 in this paper. For the two-step sample, LiNiO_2_ and Li_2_MnO_3_ were first combined in a 60:20 molar ratio. The mixture was subjected to the same mechanochemical treatment as that used for the single-step sample, and the resulting material was then sintered at 900 °C for 10 h. After grinding and vacuum-drying at 120 °C overnight, the resulting Li_1.11_Ni_0.67_Mn_0.22_O_2_ compound was mixed with Li_2_SO_4_ in an 80:20 molar ratio and then subjected to the same mechanochemical treatment. Finally, the milled product was heat-treated at 300 and 400 °C for 1 h. The final product is denoted as 80Li_1.11_Ni_0.67_Mn_0.22_O_2_·20Li_2_SO_4_ or LNMS-82 in this paper. The ratios of LiNiO_2_, Li_2_MnO_3_, and Li_2_SO_4_ were the same in both samples.

### Solid-State Battery Cell Assembly

All experiments were performed in an Ar-filled glovebox. An argyrodite-type Li_6–*x*_PS_5–*x*_Cl_1+*x*_ solid electrolyte (LPSC, Mitsui Kinzoku) was chosen as the composite positive electrode because of its high ionic conductivity (>10^–3^ S/cm at 25 °C). The composite positive electrode was prepared by mixing the active material, LPSC, and vapor-grown carbon fibers (VGCF, Resonac) in a 83.4:15.5:1.1 weight ratio, using an agate mortar for thorough blending. The resulting mixture was then incorporated into solid-state cells for the following design: [LNMS–LPSC–VGCF/LPSC/Li–In]. To construct the positive electrode layer, the composite positive electrode (8–10 mg) and the LPSC (120 mg) were placed in a polycarbonate tube (10 mm in diameter) and compressed at 720 MPa. Separately, Li–In alloys were obtained by stacking In foil (300 μm thick, 8 mm in diameter) and Li foil (250 μm thick, 6 mm in diameter), allowing both Li and In to extend onto the LPSC surface. Three-layer pellets were formed by pressing at 36 MPa and were then sandwiched between two stainless-steel disks serving as current collectors.

### Electrochemical Measurements and Materials Characterization

Charge–discharge measurements were carried out at 25 °C using a BTS2004 system (Nagano Co., Japan) at a constant current density of 0.13–2.5 mA/cm^2^ within a voltage range of 1.4–4.0 V vs Li–In, corresponding to 2.0–4.6 V vs Li. Charge–discharge measurements in a transmission electron microscope were performed using a potentio/galvanostat (SP-200, BioLogic Science Instruments) within a voltage range of 1.4–4.0 V vs Li–In. XRD analysis was carried out in the fluorescence reduction mode using a SmartLab diffractometer (Rigaku Corporation, Japan) equipped with a Cu Kα source (45 kV, 200 mA).

### Solid-State Battery Cell Assembly for In Situ STEM-EELS

All experiments were performed in an Ar-filled glovebox. As the solid electrolyte, we employed commercially available 70Li_2_S·30P_2_S_5_ glass ceramic (LPS, MSE Supplies LLC), which exhibits an ionic conductivity of ∼1.5 × 10^–3^ S/cm at room temperature. To fabricate the separator layer, 50 mg of LPS powder was compressed in a cylindrical die to form a disc with a diameter of 10 mm. LNMS particles were then lightly spread over one side of the pellet surface. The assembly was subsequently consolidated at 120 °C and 300 MPa. On the opposite face, a trilayer of In–Li–In with a thickness of 150 μm was placed (Figure S6). Finally, the resulting LNMS/LPS/Li–In stack was released from the die and directly transferred into a sputtering chamber, allowing the deposition of the Pt current collector onto the LNMS side without exposure to ambient air. The sample was cut into small fragments measuring ∼1 mm × 1 mm with a razor blade, and Cu electrodes were attached to both sides with solvent-free silver paste.

### In Situ STEM-EELS

Sample thinning for the STEM observations was performed using a focused ion-beam (FIB) system (FB-2100, Hitachi High-Tech. Corp.) operating at 40 kV that milled a section of the positive electrode layer to a thickness of ∼100 nm. STEM observations were conducted using a 300 kV transmission electron microscope (JEM-ARM300F2 GRAND ARM2, JEOL Ltd.) equipped with an EEL spectrometer (Gatan imaging filter Continuum-K3, Gatan Inc.). The prepared specimens were loaded onto a vacuum-transfer TEM holder equipped with two biasing electrodes (Mel-Build Co.). All sample transfers were performed under either inert Ar atmosphere or vacuum. EEL spectra were recorded with a dispersion of 0.15 eV/channel for the Li–K edge and 0.3 eV/channel for the O–K and Ni−L edges, using a beam current of ∼71 pA. The dwell time was 5 ms/pixel (Figure 6) or 20 ms/pixel (Figures 3 and 5) for the Li–K edge and 100 ms/pixel (Figures 3 and 5) for the O–K and Ni−L edges. The spatial step size was 1 nm (Figure 6) or 10 nm (Figures 3 and 5) for Li–K mapping and 10 nm (Figures 3 and 5) for O–K and Ni−L mappings. Figure 6 was processed in a Digital Micrograph using a 5 × 5 medium low-pass convolution filter to suppress noise and enhance feature visibility. All spectra were background-subtracted using a power-law fit. For Ni−L_3_ peak energy mapping, the Ni−L_3_ white line in each spectrum was fitted with a single Gaussian function, and the centroid of that fit was used as the peak energy across the field of view.

### Precession Electron Diffraction and Crystal Orientation Mapping

Crystal orientation maps and precession electron diffraction patterns were obtained using scanning precession electron diffraction. A nanometer-scale probe with a small convergence angle was raster-scanned across the specimen while being precessed about the optic axis, which suppressed dynamical-diffraction effects and Kikuchi lines, yielding high-quality, quasi-kinematic diffraction data at every scan point. Each precession electron diffraction pattern was compared with a library of simulated patterns covering all relevant crystal structures and orientations. The phase and orientation giving the highest correlation were assigned to the corresponding probe position. Repeating this template-matching procedure for every probe position generated crystal orientation maps for the entire scanned area. In this study, the precession angle, probe diameter, scan step size, and dwell time were 0.5°, 1–2 nm, 1 nm, and 20 ms, respectively.

## Supplementary Material



## Notes

Rate performance of solid-state batteries, X-ray diffraction patterns of LNMS-622 and LNMS-82, STEM-EDS analysis of LNMS-622, high-magnification STEM-EDS analysis of LNMS-622, precession electron diffraction of LNMS-622, experimental setup for in situ STEM-EELS, cracks formed along the grain boundaries of a LiNi_0.5_Co_0.2_Mn_0.3_O_2_ particle, STEM-EDS analysis of LNMS-82, high-magnification STEM-EDS analysis of LNMS-82, precession electron diffraction of LNMS-82, and in situ electrochemical impedance spectroscopy of the solid-state battery employing LNMS-82 performed in a transmission electron microscope (PDF)
