# Supplementary material for: Enhancing the Cycling Stability of Amorphous-Based LiNiO2–Li2MnO3–Li2SO4 Positive Electrodes: Insights from In Situ Transmission Electron Microscopy
Source: ACS Nano. 2025 Nov 24;19(48):41396–406. doi: 10.1021/acsnano.5c17388 (PMC12874628; doi:10.1021/acsnano.5c17388)
Supplement: Supplementary file 1 [file nn5c17388_si_001.pdf]

Supporting Information

Enhancing the Cycling Stability of Amorphous-Based $LiNiO_2$–Li_2MnO_3–Li_2SO_4 Positive Electrodes: Insights from In Situ Transmission Electron Microscopy

Yuki Nomura,[1,] Daiki Hiraoka,[2] Kazuo Yamamoto,[1] Tsukasa Hirayama,[1] Kota Motohashi,[2] Atsushi Sakuda,[2,*] and Akitoshi Hayashi[2]*

[1]Nanostructures Research Laboratory, Japan Fine Ceramics Center, 2–4–1 Mutsuno, Atsuta-ku, Nagoya, Aichi, 456–8587, Japan.

[2]Department of Applied Chemistry, Graduate School of Engineering, Osaka Metropolitan University, 1–1 Gakuen-cho, Naka-ku, Sakai, Osaka 599-8531, Japan.

* To whom correspondence should be addressed. E-mail: y_nomura@jfcc.or.jp, saku@omu.ac.jp

Table of Contents

Fig. S1. Rate performance of solid-state batteries.

Fig. S2. X-ray diffraction patterns of LNMS-622 and LNMS-82.

Fig. S3. STEM-EDS analysis of LNMS-622.

Fig. S4. High-magnification STEM-EDS analysis of LNMS-622.

Fig. S5. Precession electron diffraction of LNMS-622.

Fig. S6. Experimental setup for in situ STEM-EELS.

Fig. S7. Cracks formed along the grain boundaries of a $LiNi_{0.5}Co_{0.2}Mn_{0.3}O_2$ particle.

Fig. S8. STEM-EDS analysis of LNMS-82.

Fig. S9. High-magnification STEM-EDS analysis of LNMS-82.

Fig. S10. Precession electron diffraction of LNMS-82.

Fig. S11. In situ electrochemical impedance spectroscopy of the solid-state battery employing LNMS-82 performed inside a transmission electron microscope.

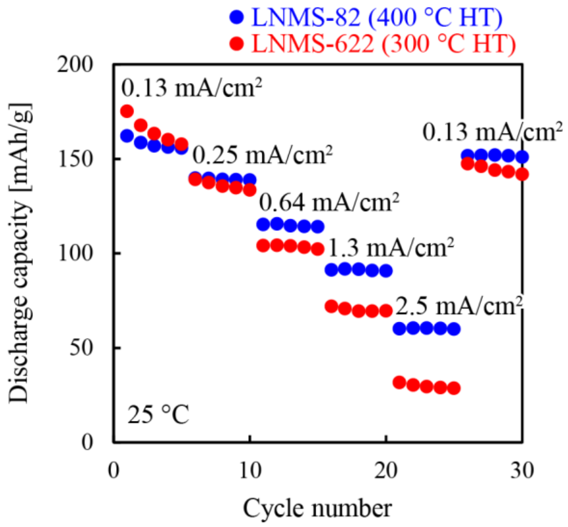

Figure S1. Rate performance of solid-state batteries incorporating $60LiNiO_2 \cdot 20Li_2MnO_3 \cdot 20Li_2SO_4$ (LNMS-622, prepared via a single-step process) and $80Li_{1.11}Ni_{0.67}Mn_{0.22}O_2 \cdot 20Li_2SO_4$ (LNMS-82, prepared via a two-step process).

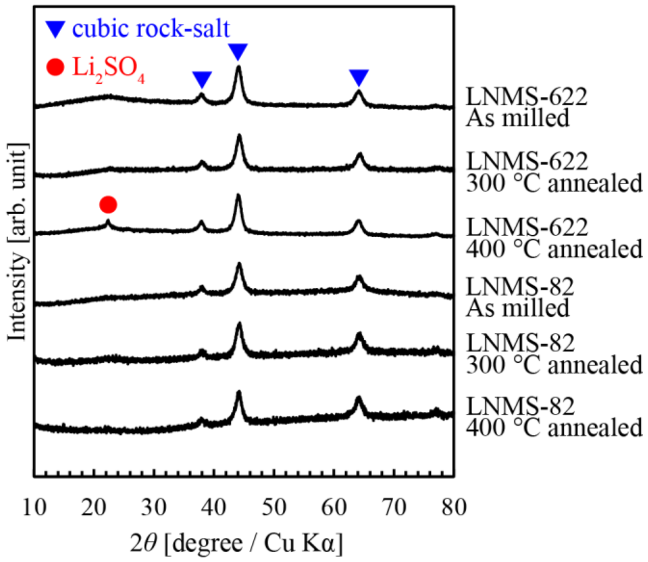

Figure S2. X-ray diffraction patterns of LNMS-622 and LNMS-82 after the mechanochemical treatment followed by heat treatment at 300 or 400 °C.

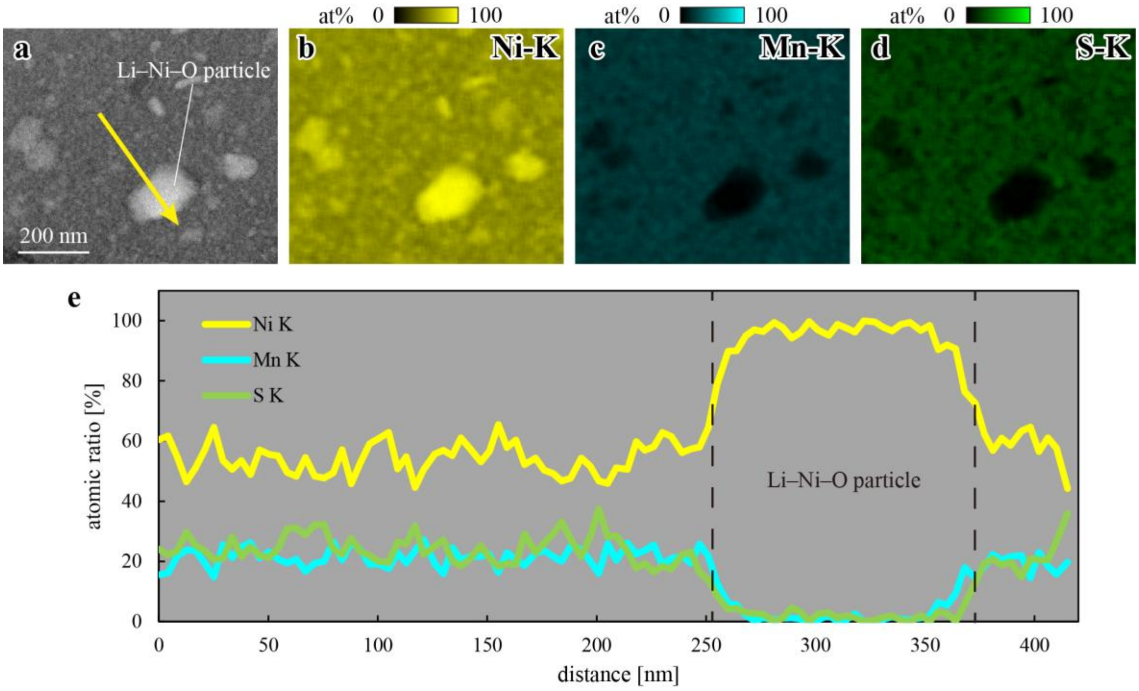

Figure S3. STEM-EDS analysis of LNMS-622. (a) ADF-STEM image. (b–d) Quantitative elemental ratio maps of Ni, Mn, and S. (e) Line profiles of atomic ratios of Ni, Mn, and S.

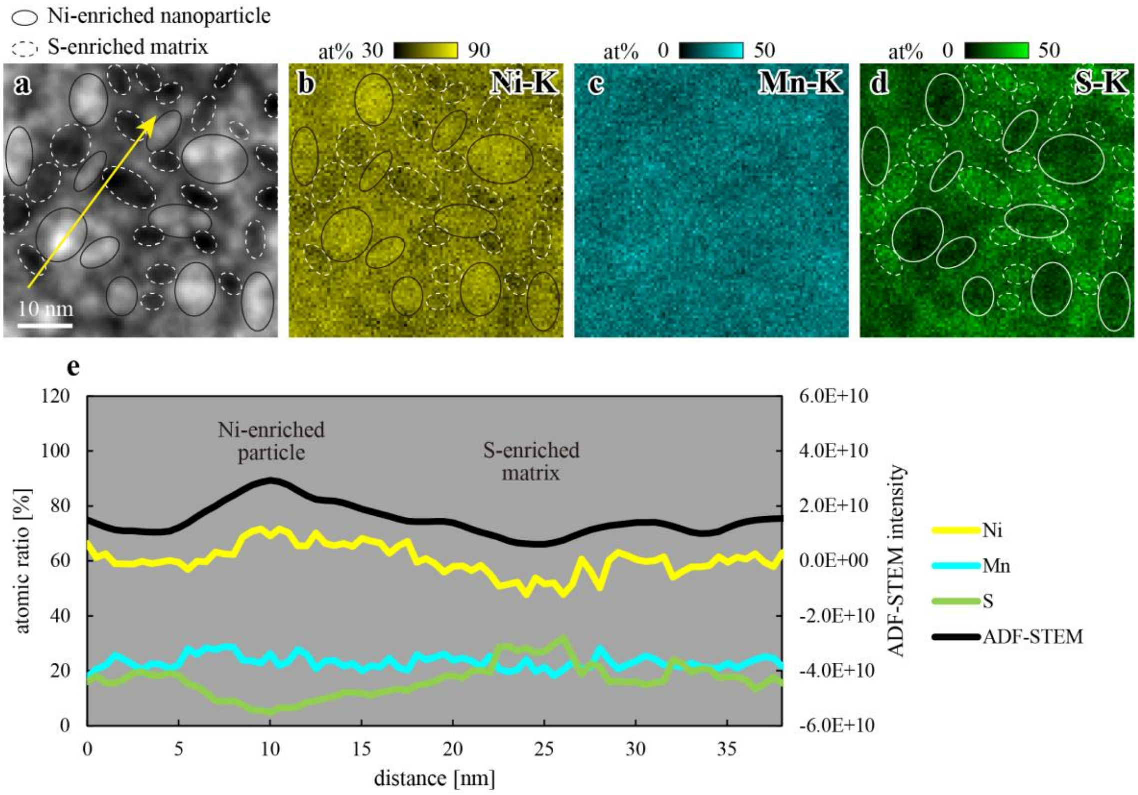

Figure S4. High-magnification STEM-EDS analysis of LNMS-622. (a) ADF-STEM image. (b–d) Quantitative elemental ratio maps of Ni, Mn, and S. (e) Line profiles of atomic ratios of Ni, Mn, and S.

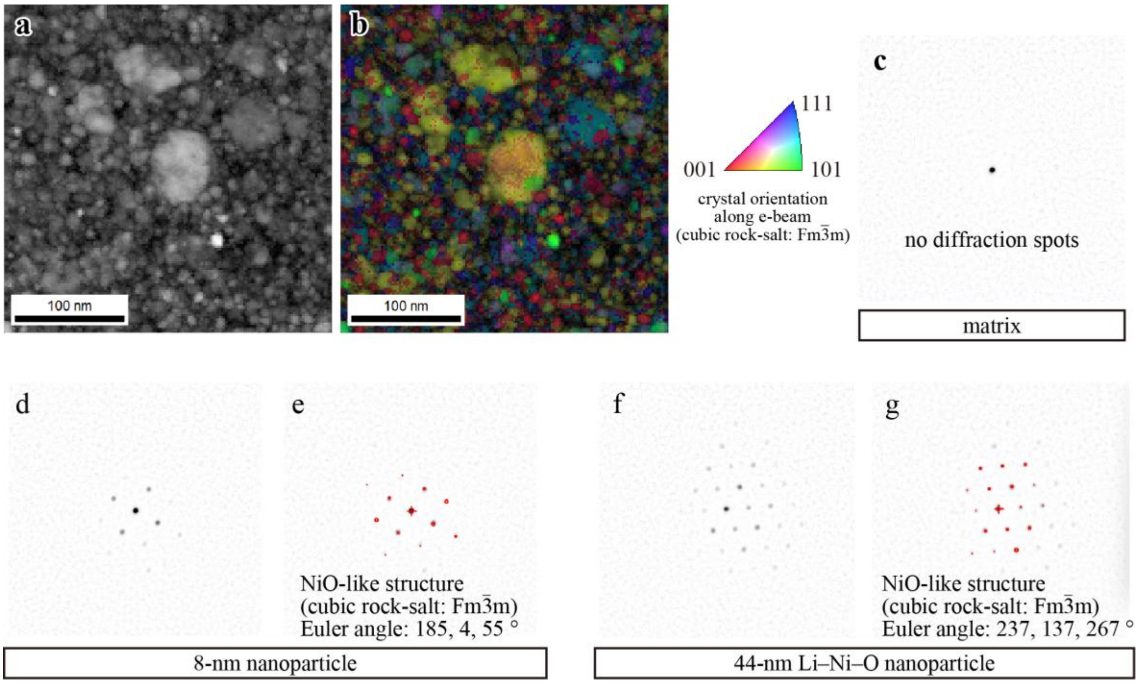

Figure S5. Precession electron diffraction of LNMS-622. (a) Image-quality map. (b) Crystal orientation map of crystalline domains along the electron beam. (c) Experimental precession electron diffraction patterns from the S-enriched matrix, (d) 8-nm Ni-enriched nanoparticle, and (f) 44-nm Li–Ni–O nanoparticle. (e, g) Corresponding matched templates.

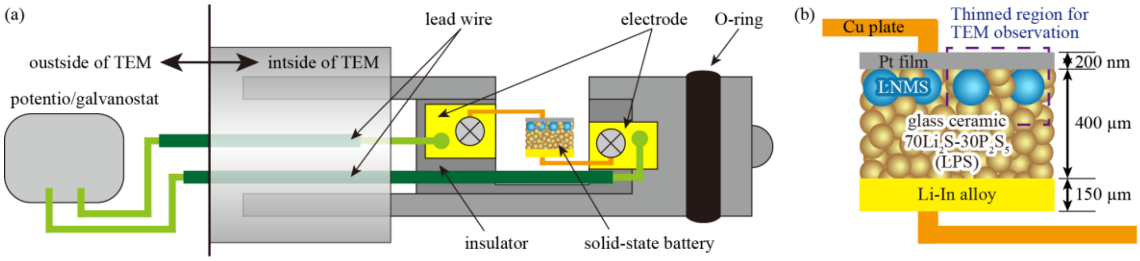

Figure S6. Experimental setup for in situ STEM-EELS. (a) Schematic of the inert/vacuum-transfer biasing TEM holder. (b) Schematic cross-section of the bulk-type solid-state battery: a $70Li_2S{\cdot}30P_2S_5$ glass ceramic (LPS) is used as the solid electrolyte, Li-In alloy film serves as the negative electrode, and Pt film is sputtered onto LNMS particles as the current collector. The dashed purple rectangle indicates the FIB-thinned region prepared for TEM observation.

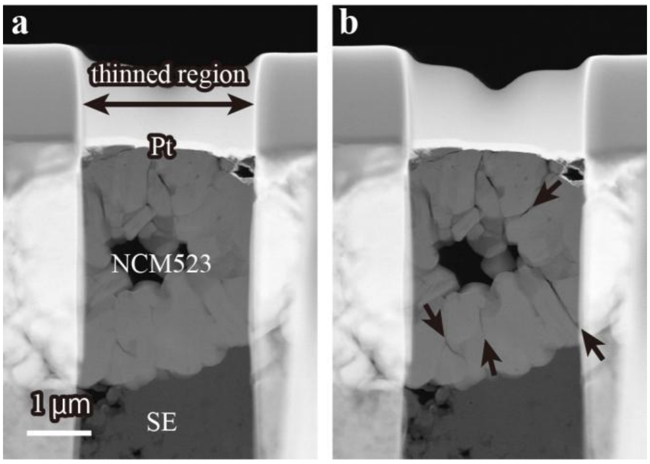

Figure S7. Cracks formed along the grain boundaries of a $LiNi_{0.5}Co_{0.2}Mn_{0.3}O_2$ particle. (a) ADF-STEM image acquired before charging. (b) ADF-STEM image acquired after charging to 3.9 V vs. Li-In (4.5 V vs. Li), showing the development of cracks (arrows) along the grain boundaries.

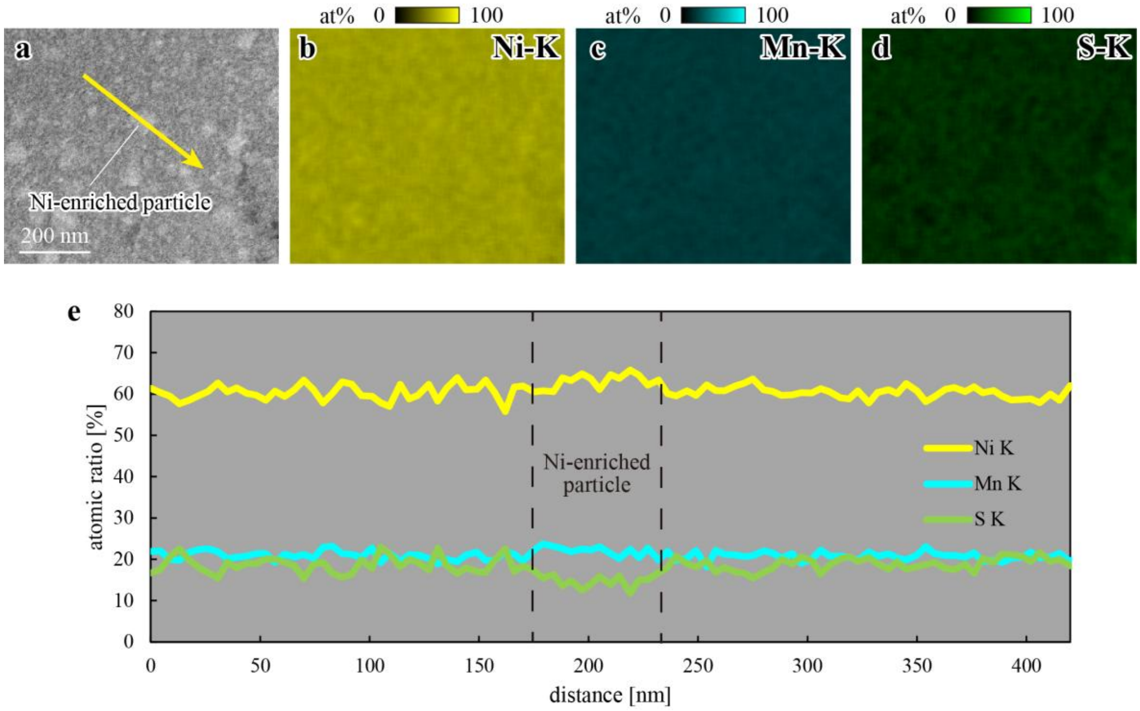

Figure S8. STEM-EDS analysis of LNMS-82. (a) ADF-STEM image. (b–d) Quantitative elemental ratio maps of Ni, Mn, and S. (e) Line profiles of atomic ratios of Ni, Mn, and S.

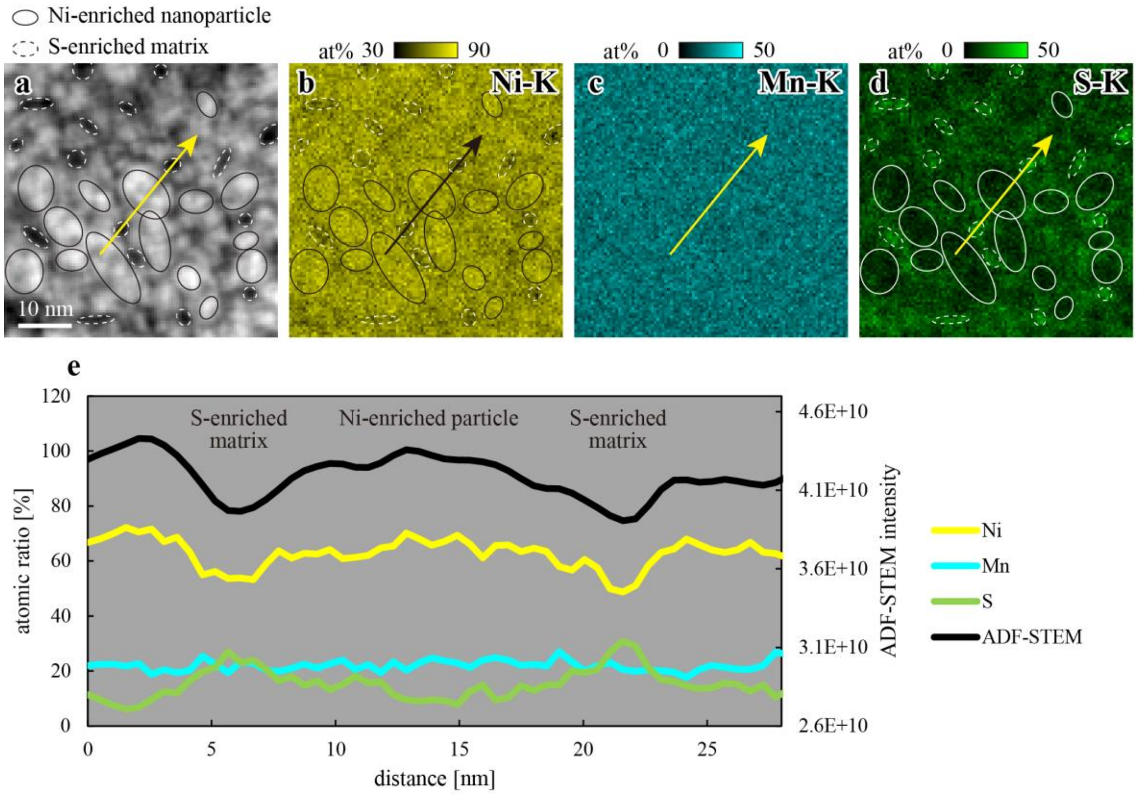

Figure S9. High-magnification STEM-EDS analysis of LNMS-82. (a) ADF-STEM image. (b–d) Quantitative elemental ratio maps of Ni, Mn, and S. (e) Line profiles of atomic ratios of Ni, Mn, and S.

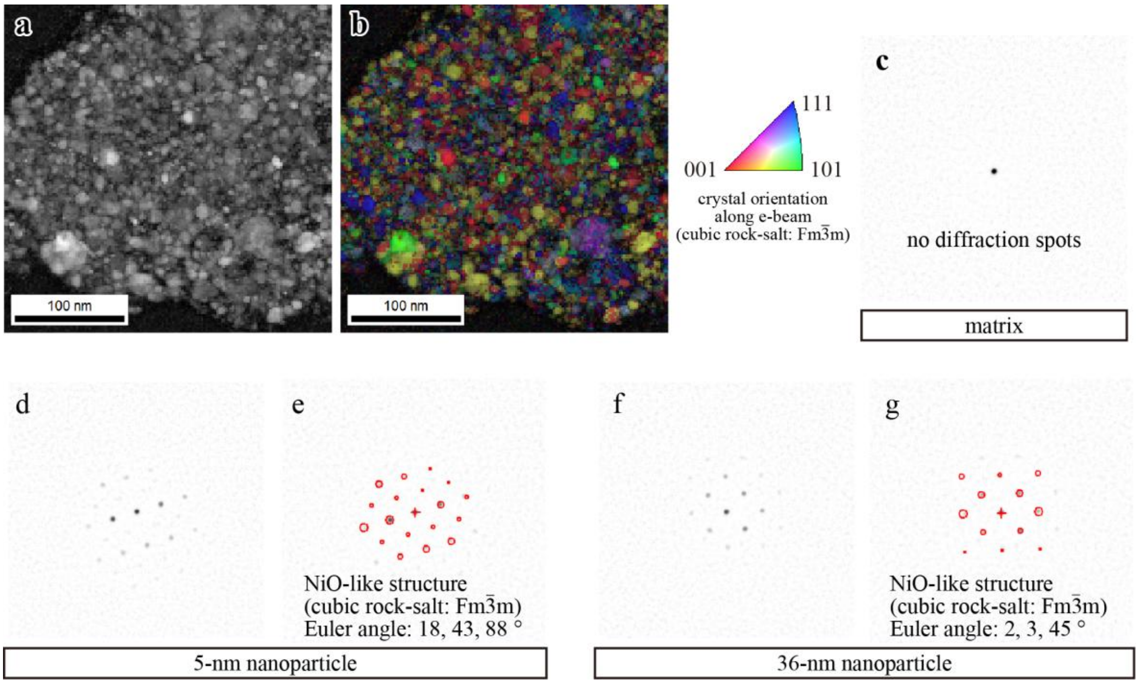

Figure S10. Precession electron diffraction of LNMS-82. (a) Image-quality map. (b) Crystal orientation map of crystalline domains along the electron beam. (c) Experimental precession electron diffraction patterns from the S-enriched matrix, (d) 5-nm Ni-enriched nanoparticle, and (f) 36-nm Ni-enriched nanoparticle. (e, g) Corresponding matched templates.

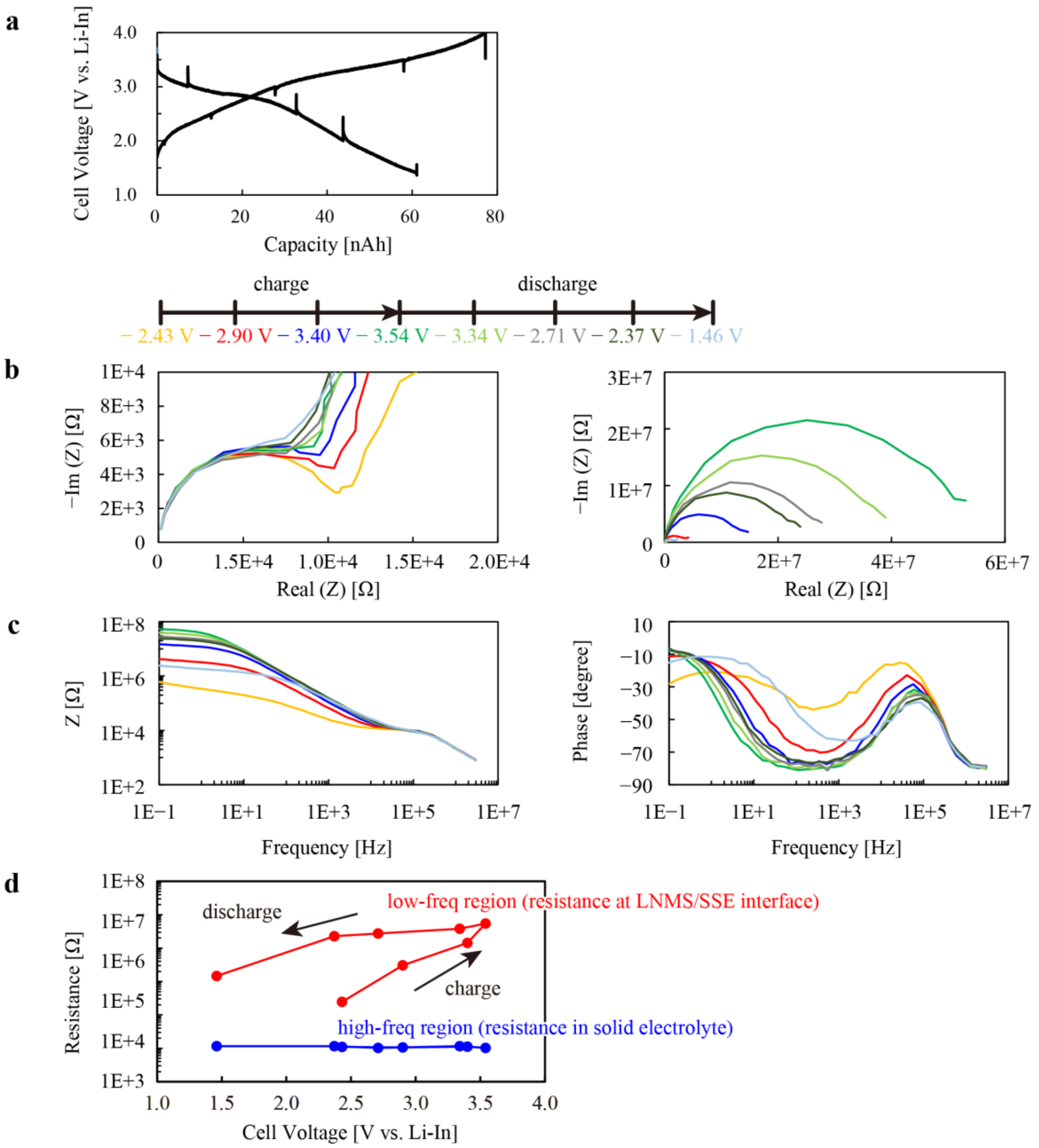

Figure S11. In situ electrochemical impedance spectroscopy of the solid-state battery employing LNMS-82 performed inside a transmission electron microscope. (a) First charge-discharge curves. (b, c) Nyquist (b) and Bode (c) plots recorded at various states of charge. (d) Evolution of the two resistances obtained by fitting the impedance spectra.
